# Supplementary material for: CXCL4 synergizes with TLR8 for TBK1-IRF5 activation, epigenomic remodeling and inflammatory response in human monocytes
Source: Nat Commun. 2022 Jun 14;13:3426. doi: 10.1038/s41467-022-31132-7 (PMC9195402; doi:10.1038/s41467-022-31132-7)
Supplement: Supplementary file 1 — Supplementary Information [file 41467_2022_31132_MOESM1_ESM.pdf]

CXCL4 synergizes with TLR8 for TBK1-IRF5 activation, epigenomic remodeling and inflammatory response in human monocytes

Chao Yang[1], Mahesh Bachu[1], Yong Du[1], Caroline Brauner[1], Ruoxi Yuan[1], Marie Dominique Ah Kioon[1], Giancarlo Chesi[1], Franck J. Barrat[1,2,3], Lionel B. Ivashkiv[1,2,4*]

[1]HSS Research Institute and David Z. Rosensweig Genomics Research Center, Hospital for Special Surgery, New York, NY; [2]Immunology and Microbial Pathogenesis Program and [3]Department of Microbiology and Immunology and [4]Department of Medicine, Weill Cornell Medicine, New York, NY.

[*] Correspondence: IvashkivL@hss.edu

Supplementary figures: 15

Supplementary table 1

Supplementary table 2

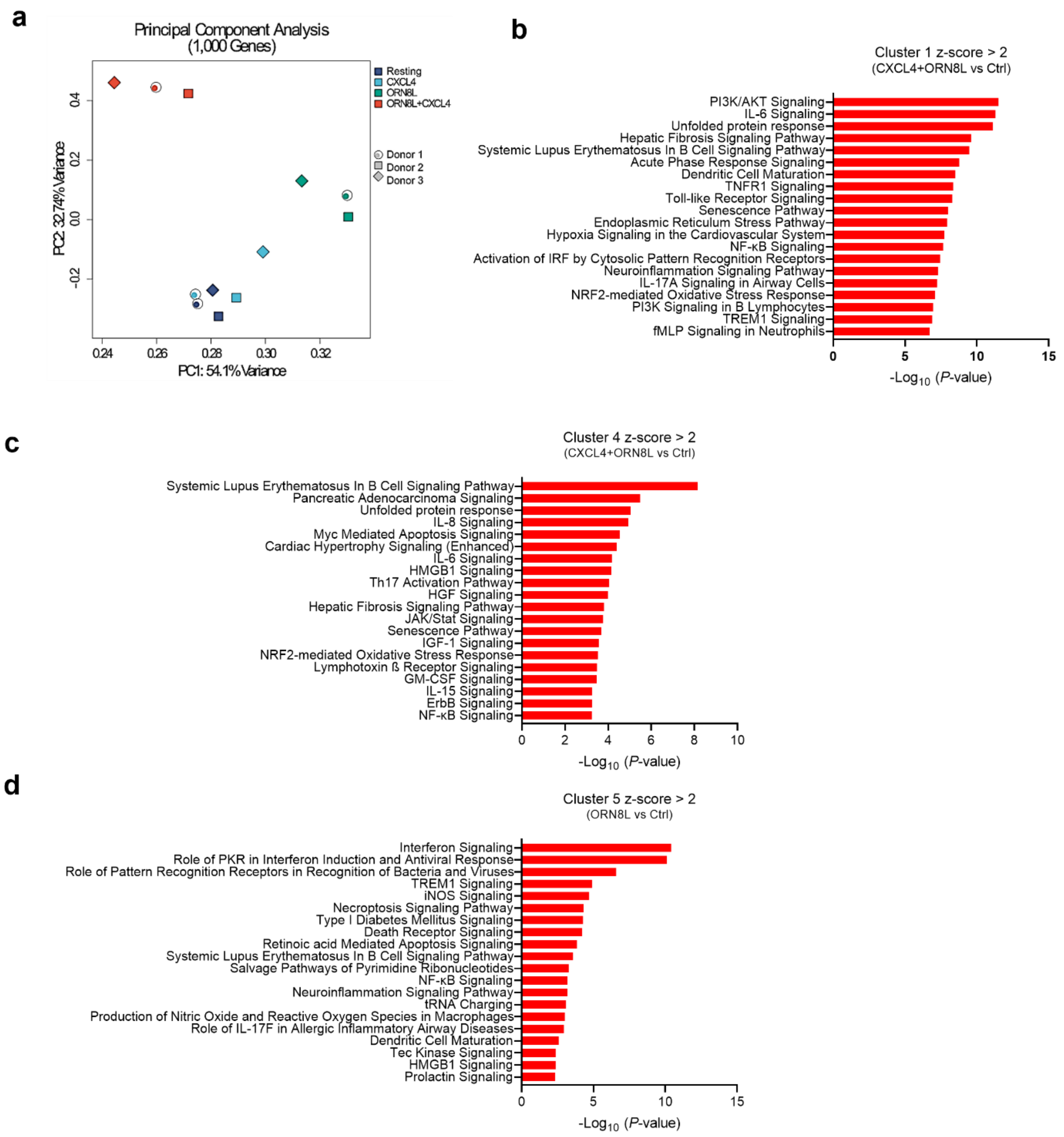

Supplementary Figure 1. (**a**) Principal Component Analysis (PCA) of RNAseq data. (**b - d**) IPA pathway analysis of gene clusters from **Figure 1a.** RNAseq was performed with three independent biological replicates.

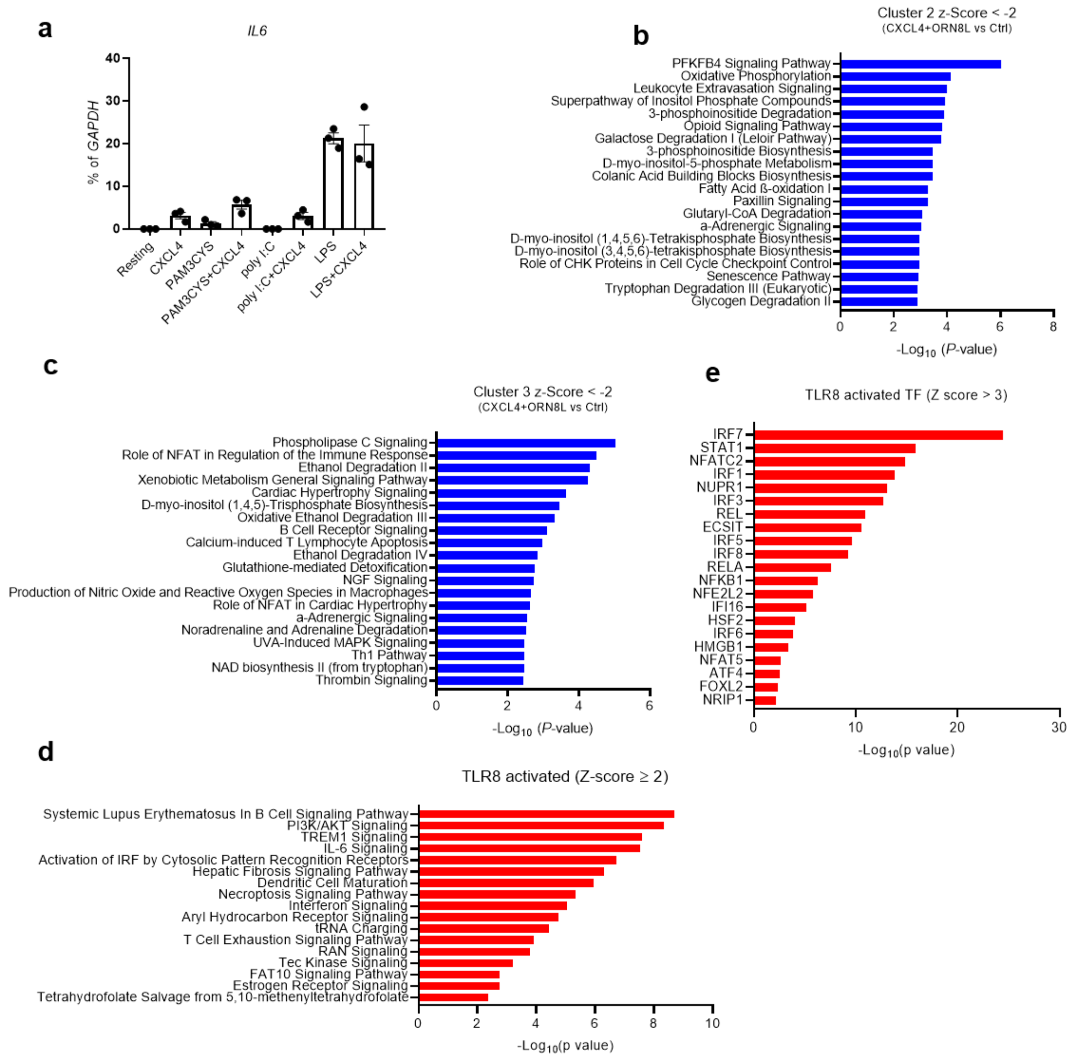

Supplementary Figure 2. (**a**) mRNA of *IL6* was measured by quantitative PCR (qPCR) and normalized relative to *GAPDH* mRNA. n = 3 independent experiments. (**b, c**) IPA pathway analysis of gene clusters 2 and 3 from **Figure 1a**. (**d, e**) IPA pathway and TF analysis of genes upregulated by ORN8L from figure 1a. Data is depicted as mean ± SEM for **c**. Source data are provided as a Source Data file.

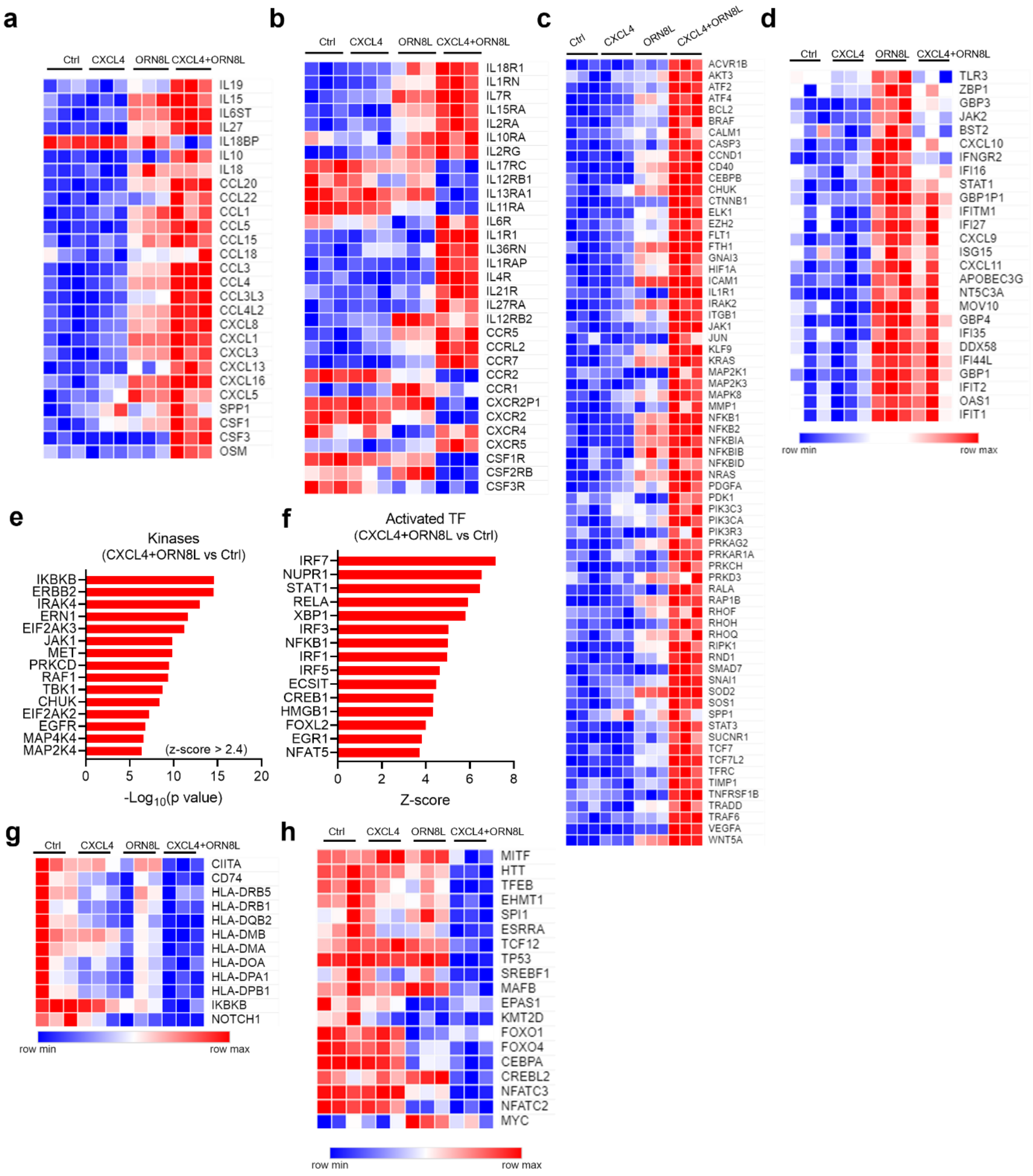

Supplementary Figure 3. (**a - d, g, h**) Heatmaps showing expression of representative cytokine and chemokine genes (**a**) and their receptors (**b**) and fibrosis related genes (**c**), and ISGs (**d**), and antigen presentation related genes (**g**) and transcription factors important for anabolic metabolism and osteoclastogenesis (**h**) assessed in experimental condition as in **Figure 1a** and presented (key) relative to the maximum. (**e, f**) IPA activated kinases and TF analysis of genes upregulated by CXCL4 + ORN8L from **figure 1a**.

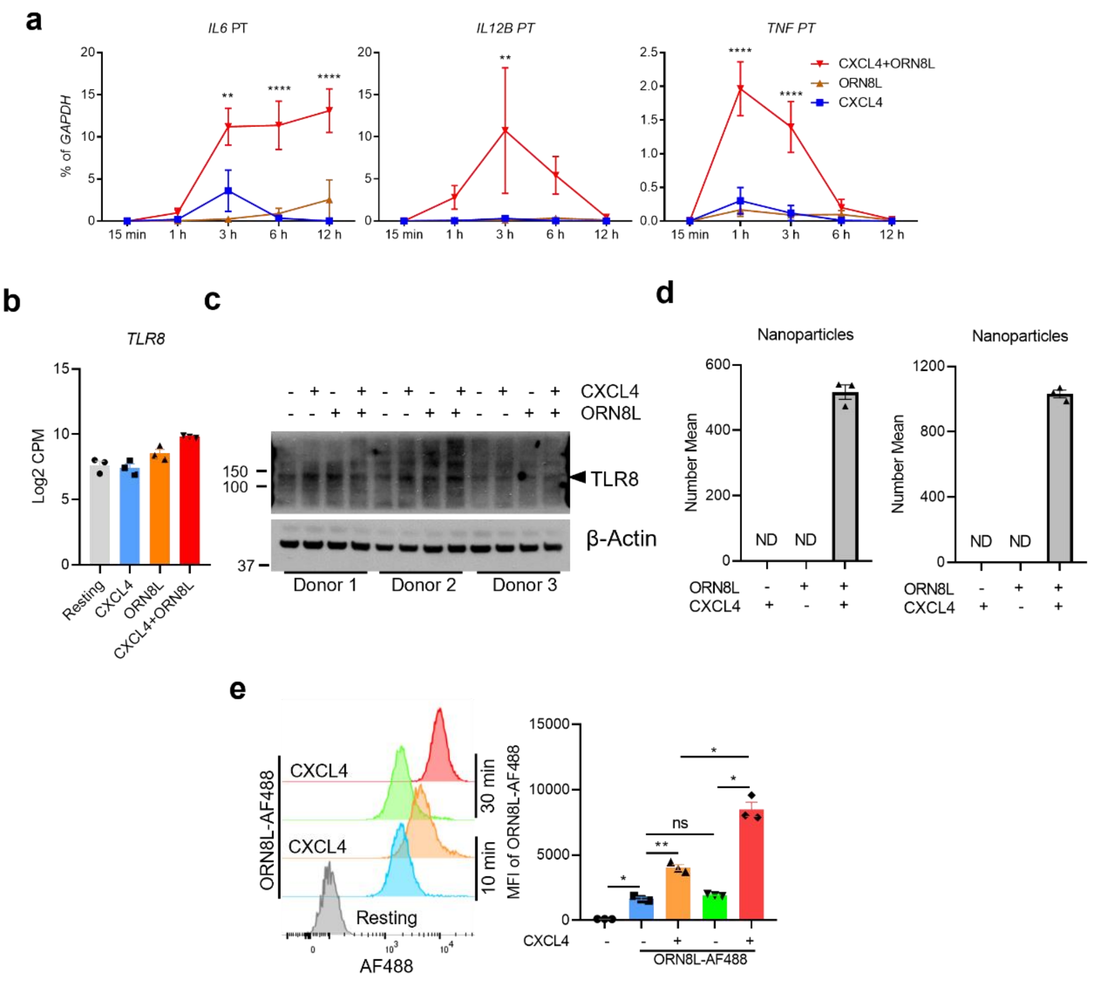

Supplementary Figure 4. (**a**) Primary transcripts (PT) of *IL6*, *IL12B* and *TNF* were measured by quantitative PCR (qPCR) and normalized relative to *GAPDH* mRNA (n = 4 donors). (**b**) Log2 CPM of TLR8 from RNAseq data in human monocytes 6 hr after CXCL4 and/or ORN8L stimulation. (**c**) Immunoblots of TLR8 using whole cell lysates from the indicated conditions 6 hr after stimulation (data are representative of 3 independent experiments). (**d**) Nanoparticle formation measured using dynamic light scattering. The number mean is from one sample measured in triplicate. ND = not detected. 2 independent experiments are shown to supplement the third experiment shown in Fig. 1f. (**e**) Flow cytometric analysis of the internalization of ORN8L-AF488 after the indicated times of incubation in the absence or presence of CXCL4 in human monocytes. Left panel, representative FACS plot; right panel, cumulative data (n = 3 donors). Data is depicted as mean ± SEM; ****p ≤ 0.0001; ***p ≤ 0.001; **p ≤ 0.01; *p ≤ 0.05 by two-way ANOVA (**a**) or one-way ANOVA (**e**). Source data are provided as a Source Data file.

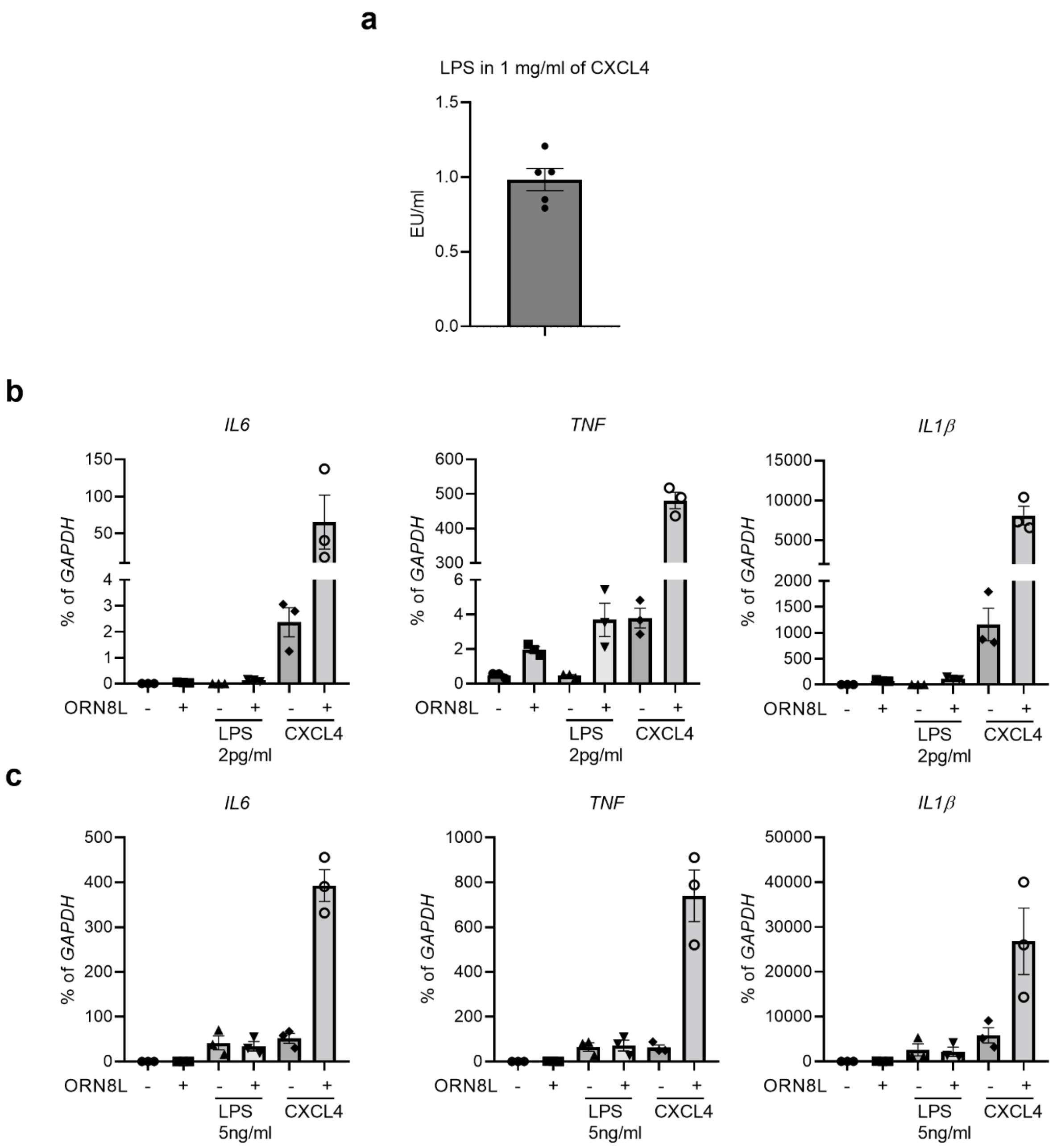

Supplementary Figure 5. (**a**) Endotoxin assay of CXCL4 stock solution (n = 5 technical replicates). (**b** and **c**) qPCR analysis of mRNA amounts of *IL6*, *TNF* and *IL1β* normalized relative to *GAPDH* mRNA in cells stimulated with 2 pg/ml LPS (n = 3 donors) (**b**) or 5 ng/ml LPS (n = 3 donors) (**c**) in the presence/absence of ORN8L, CXCL4 serves as a positive control. Data are depicted as mean ± SEM. Source data are provided as a Source Data file.

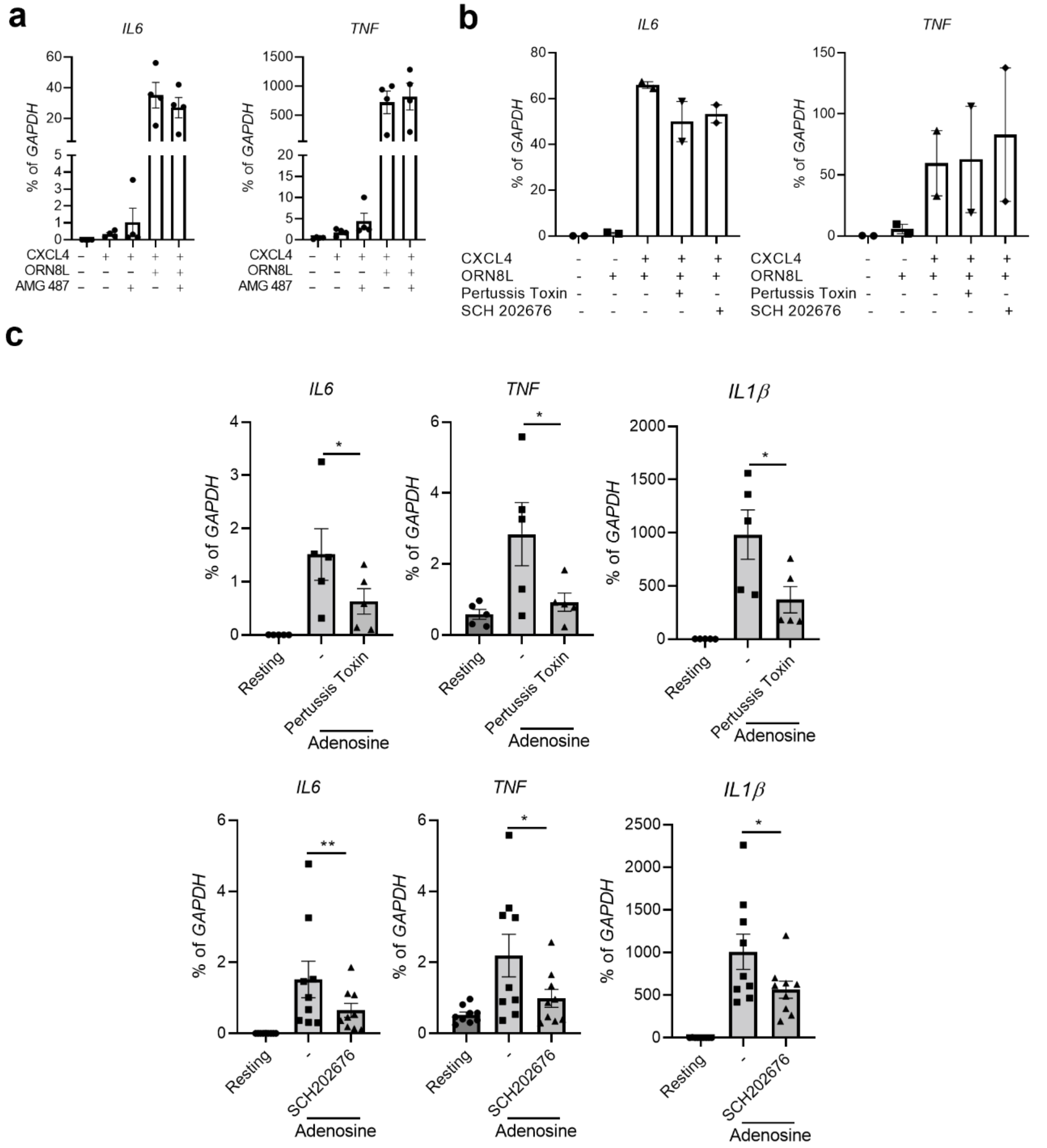

Supplementary Figure 6. (**a**) mRNA of *IL6* and *TNF* was measured by qPCR and normalized relative to *GAPDH* mRNA in cells stimulated with CXCL4 and/or ORN8L with/without CXCR3 inhibitor AMG487 (5 µM) for 3 hr (n = 4 donors). (**b** and **c**) mRNA of *IL6* and *TNF* was measured by qPCR and normalized relative to *GAPDH* mRNA in cells stimulated with CXCL4 and/or ORN8L (**b**, n = 2 donors) or adenosine (1mM) (**c**, n = (upper panels) or 9 (lower panels)) with/without G-coupled receptor inhibitors Pertussis toxin (10 µM) or SCH202675 (10 µM) for 6 h. Data depict mean ± SEM; **p ⩽ 0.01; *p ⩽ 0.05 by paired t test, Two-tailed (**c**)). Source data are provided as a Source Data file.

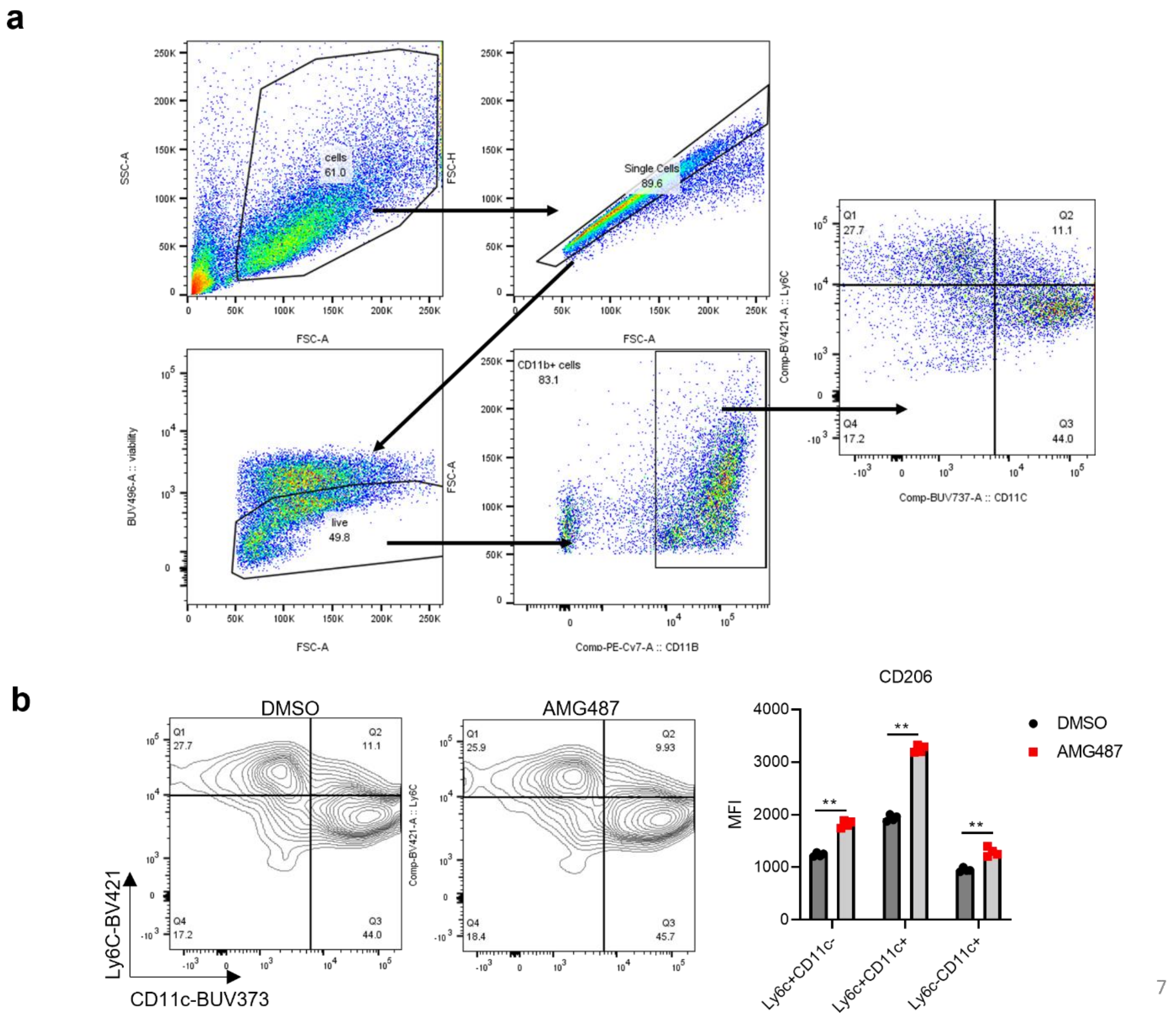

Supplementary Figure 7. (**a**) The gating strategy used for CD11b$^+$ cells and then separate the CD11b$^+$ cells into 4 different populations using CD11c and Ly6C markers. (**b**) FACS to analyze CD206 levels in Ly6C$^+$CD11c$^-$, Ly6C$^+$CD11c$^+$ and Ly6C$^-$CD11c$^+$ BMDCs with/without CXCR3 inhibitor AMG487 (5 μM) for 24 hr (n = 4 independent experiment). Data depict mean ± SEM; **p ≤ 0.01 by two-way ANOVA (**d**). Source data are provided as a Source Data file.

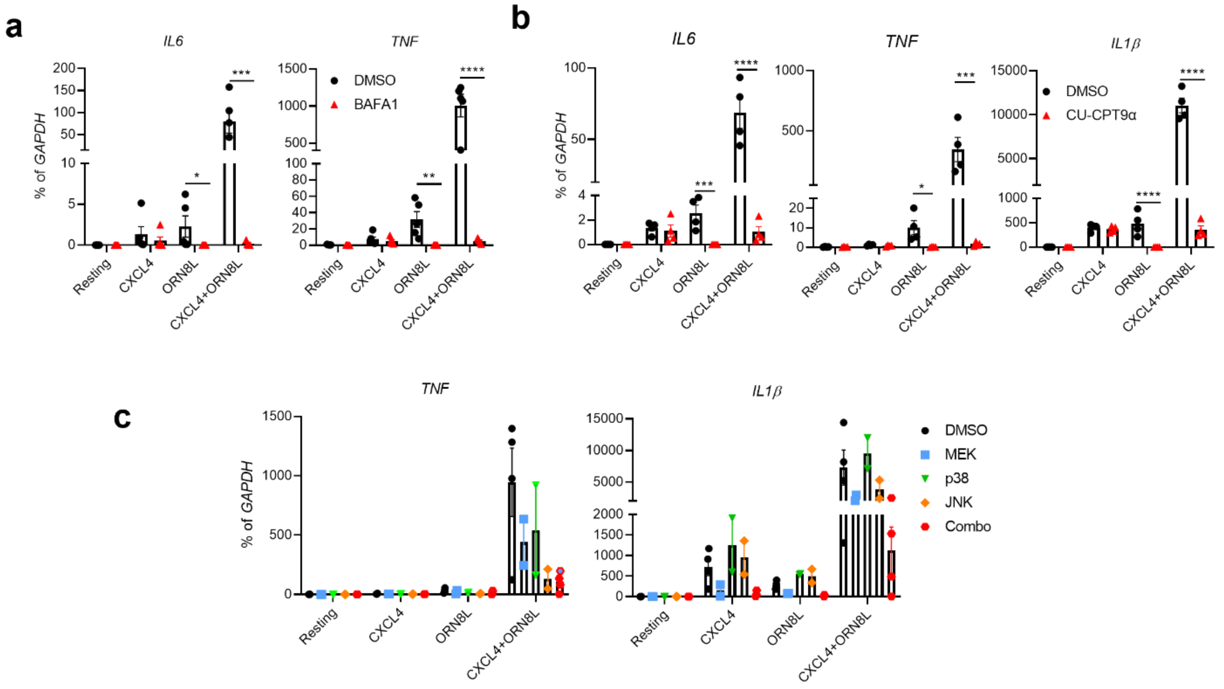

Supplementary Figure 8. (**a** and **b**) mRNA of *IL6*, *TNF* and *IL1β* was measured by qPCR and normalized relative to *GAPDH* mRNA in cells stimulated with CXCL4 and/or ORN8L with/without Bafilomycin A1 (BAFA1) (1 µM) (n = 5 donors), TLR8 inhibitor CU-CPT9α (1 µM) (n = 4 donors) for 3 h, respectively. (**c**) qPCR analysis of mRNA amounts of *TNF* and *IL1β* normalized relative to *GAPDH* mRNA in cells stimulated with CXCL4 and/or ORN8L after treatment with the MAPK inhibitors SB 202190 (p38), JNK inhibitor II and U0126 (MEK1/2) used at 10 µM, respectively (n = 4 donors). The data are related to and use some of the same samples as Fig. 2g. Data is depicted as mean ± SEMdonors; ****$p \leq 0.0001$; ***$p \leq 0.001$; **$p \leq 0.01$; *$p \leq 0.05$ by two-way ANOVA. Source data are provided as a Source Data file.

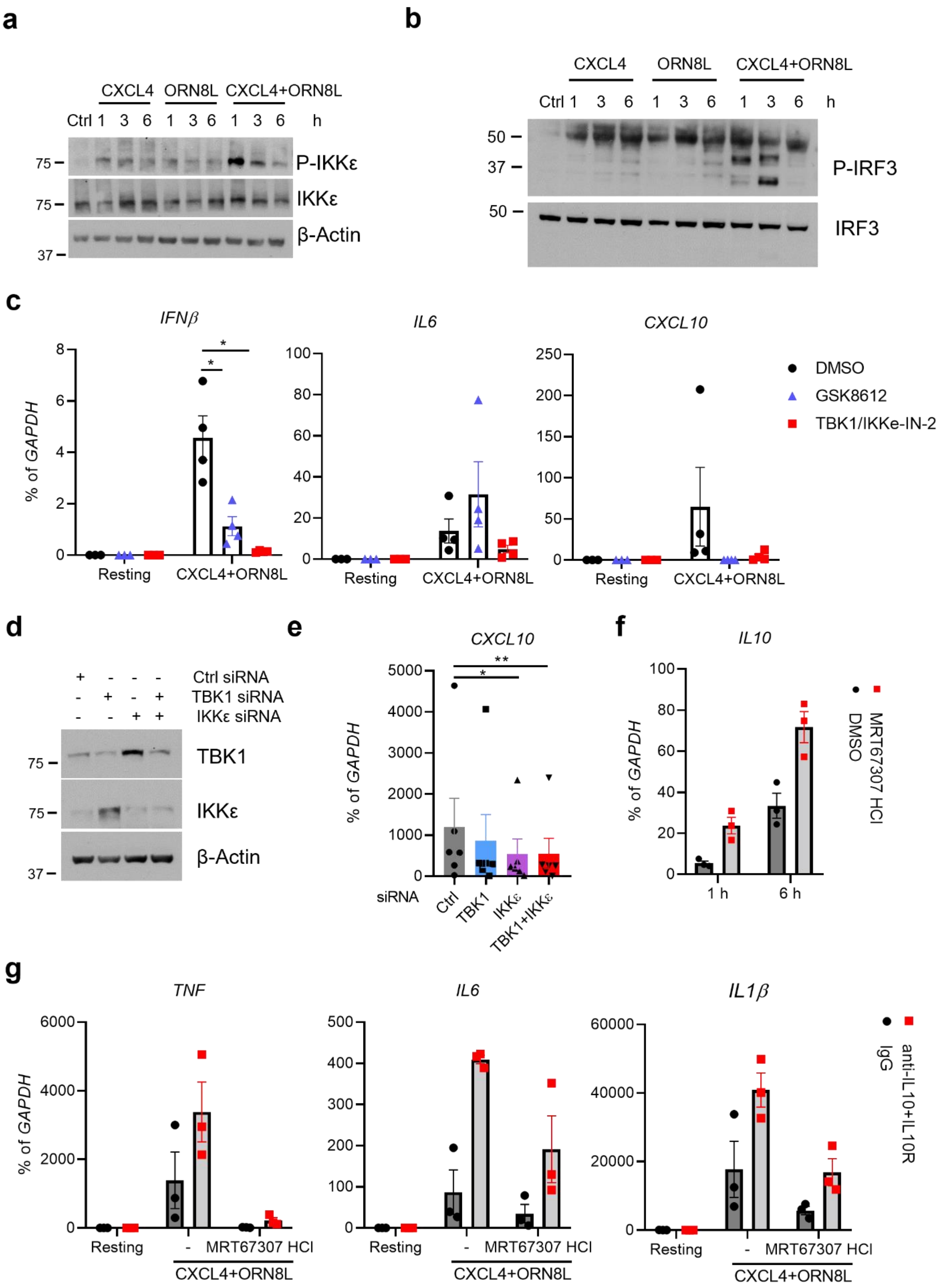

a
CXCL4
ORN8L
CXCL4+ORN8L
Ctrl 1 3 6 1 3 6 1 3 6 h
P-IKKε
IKKε
β-Actin
b
P-IRF3
IRF3
c
IFNβ
IL6
CXCL10
% of GAPDH
Resting
CXCL4+ORN8L
DMSO
GSK8612
TBK1/IKKe-IN-2
d
Ctrl siRNA
TBK1 siRNA
IKKε siRNA
TBK1
IKKε
β-Actin
e
CXCL10
siRNA
Ctrl
TBK1
IKKε
TBK1+IKKε
f
IL10
DMSO
MRT67307 HCl
1 h
6 h
g
TNF
IL6
IL1β
IgG
anti-IL10+IL10R
MRT67307 HCl

Supplementary Figure 9. (**a** and **b**) Immunoblots of phospho-IKKε, total IKKε (**a**) and phospho-IRF3 and total IRF3 (**b**) using whole cell lysates from a time course with the indicated conditions. (**c**) mRNA of indicated genes was measured by qPCR and normalized relative to *GAPDH* mRNA after blockade of TBK1/IKKε activation by 1 μM of TBK1/IKKε-IN-2 or 50 μM of GSK8612 (n = 4 independent experiments) for 3 h. (**d**) Immunoblot of TBK1 and IKKε with whole cell lysates from monocytes nucleofected with control or TBK1- and/or IKKε-specific siRNAs. (data representative of 3 independent experiments) (**e**) mRNA of *CXCL10* was measured by qPCR and normalized relative to *GAPDH* mRNA using monocytes nucleofected with control or TBK1- and/or IKKε-specific siRNAs (n = 6 donors). (**f**) mRNA level of *IL10* measured by qPCR and normalized relative to *GAPDH* mRNA (n = 3 independent experiments). (**g**) mRNA of indicated genes was measured by qPCR and normalized relative to *GAPDH* mRNA after blockade of IL-10 and IL10R with/without TBK1 inhibitor MRT67307 HCl (n = 3 independent experiments). Data in (**a** and **b**) are representative of 3 experiments. Data are depicted as mean ± SEM (**c**, **e** - **g**). **p ≤ 0.01; *p ≤ 0.05 by two-way ANOVA (**c**) or by Friedman test (**e**). Source data are provided as a Source Data file.

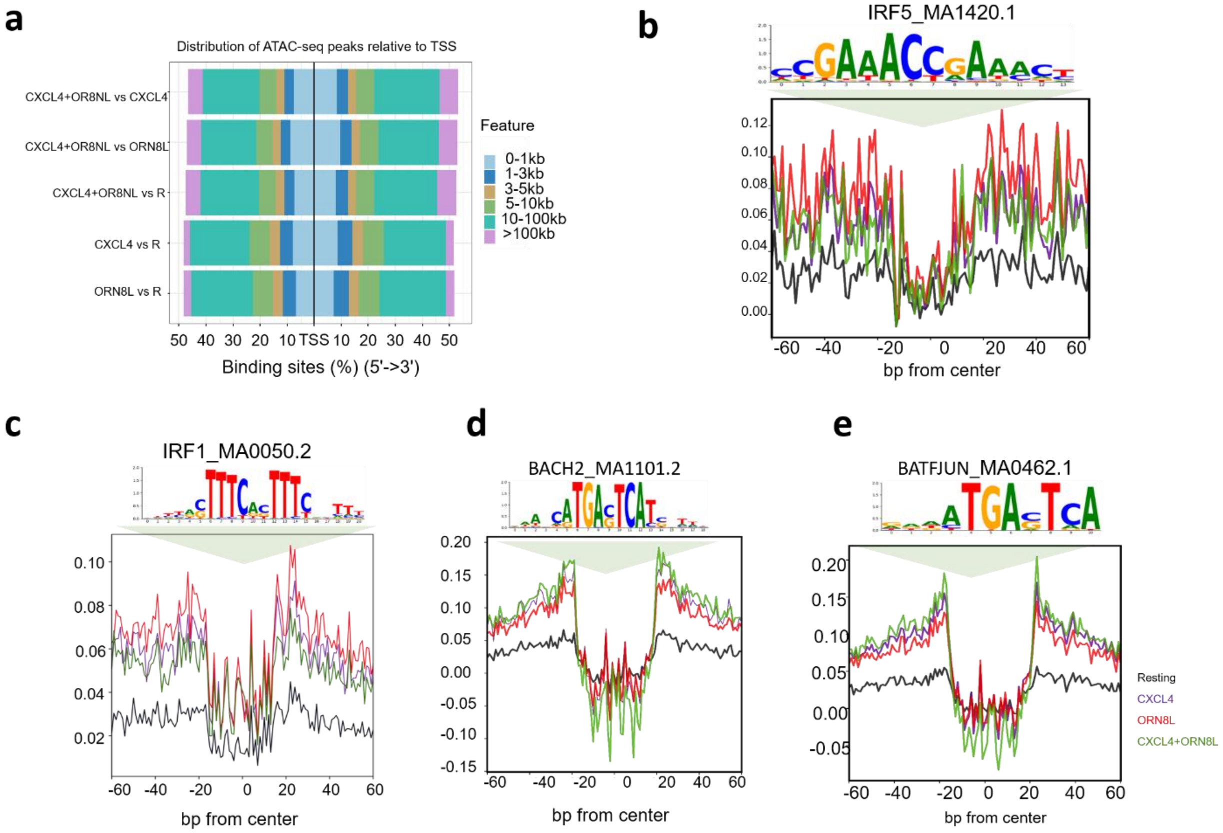

Supplementary Figure 10. (**a**) Chart representation of the relative distribution of ATAC-seq peak coordinates relative to the position of transcription start sites (TSS) for differential open chromatin regions identified by comparison of respective treatments versus the resting condition. (**b** - **e**) Visualization of significant footprints for all accessible sites for the following significant motifs IRF1_MA0050.2, IRF5_MA1420.1, BACH2_MA1101.2, BATFJUN_MA0462.1 that were identified by BINDetect using TOBIAS.

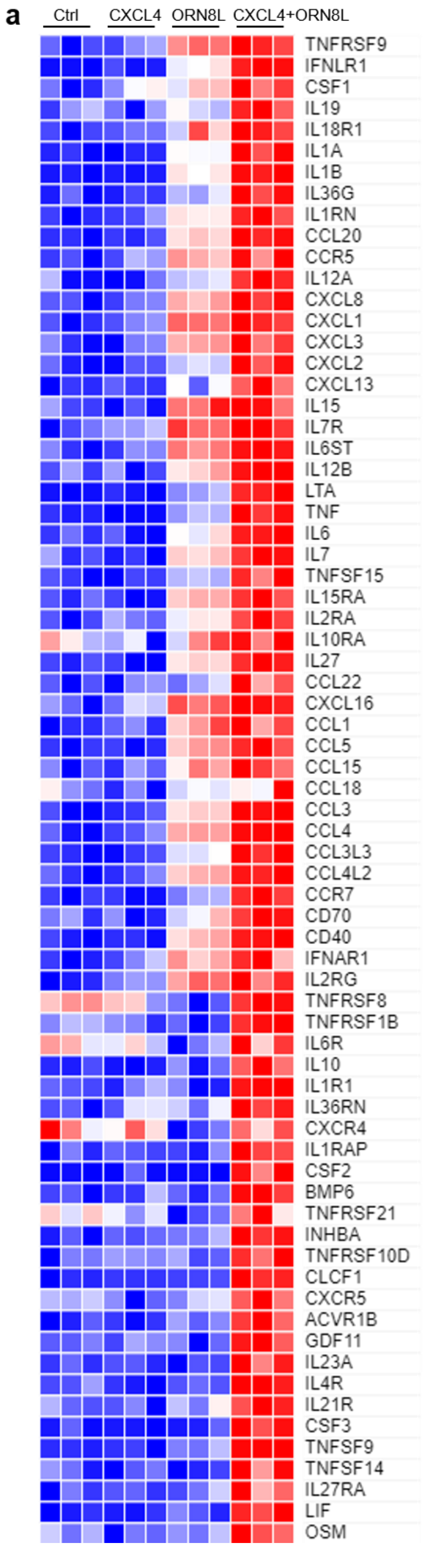

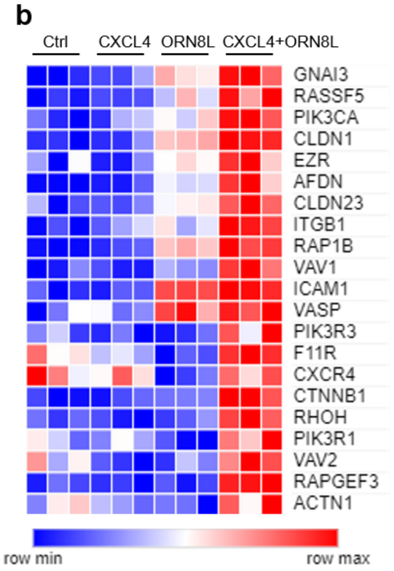

Supplementary Figure 11. (**a**) Heatmap showing representative genes associated with C1 peaks in the cytokine-cytokine receptor pathway. (**b**) Heatmap showing representative genes associated with C1 peaks in cell adhesion and migration pathways. These data are from the ATACseq experiments shown in Figs. 4 and 5; n = 3 independent experiments.

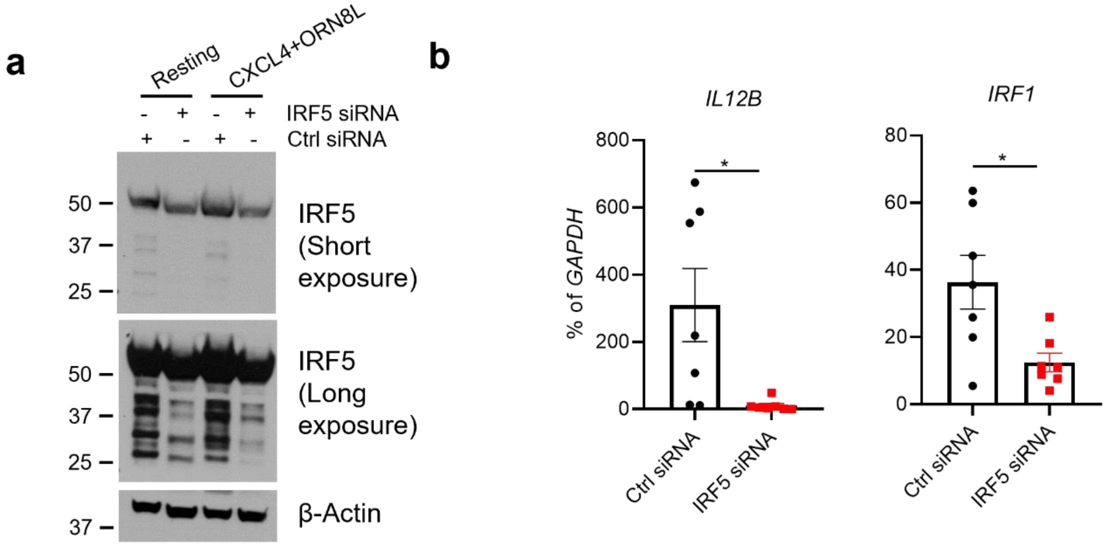

Supplementary Figure 12. (**a**) Immunoblot of IRF5 using whole cell lysates 3 days after nucleofection of monocytes with control or IRF5-specific siRNAs. (**b**) mRNA of indicated genes was measured by qPCR and normalized relative to *GAPDH* mRNA after knockdown of IRF5 by siRNA for 3 days (n = 7 independent experiments). Data are representative of 3 independent experiments (**a**) or depict mean ± SEM (**b**). *p ≤ 0.05 by Wilcoxon signed-rank test, Two-tailed (**b**). Source data are provided as a Source Data file.

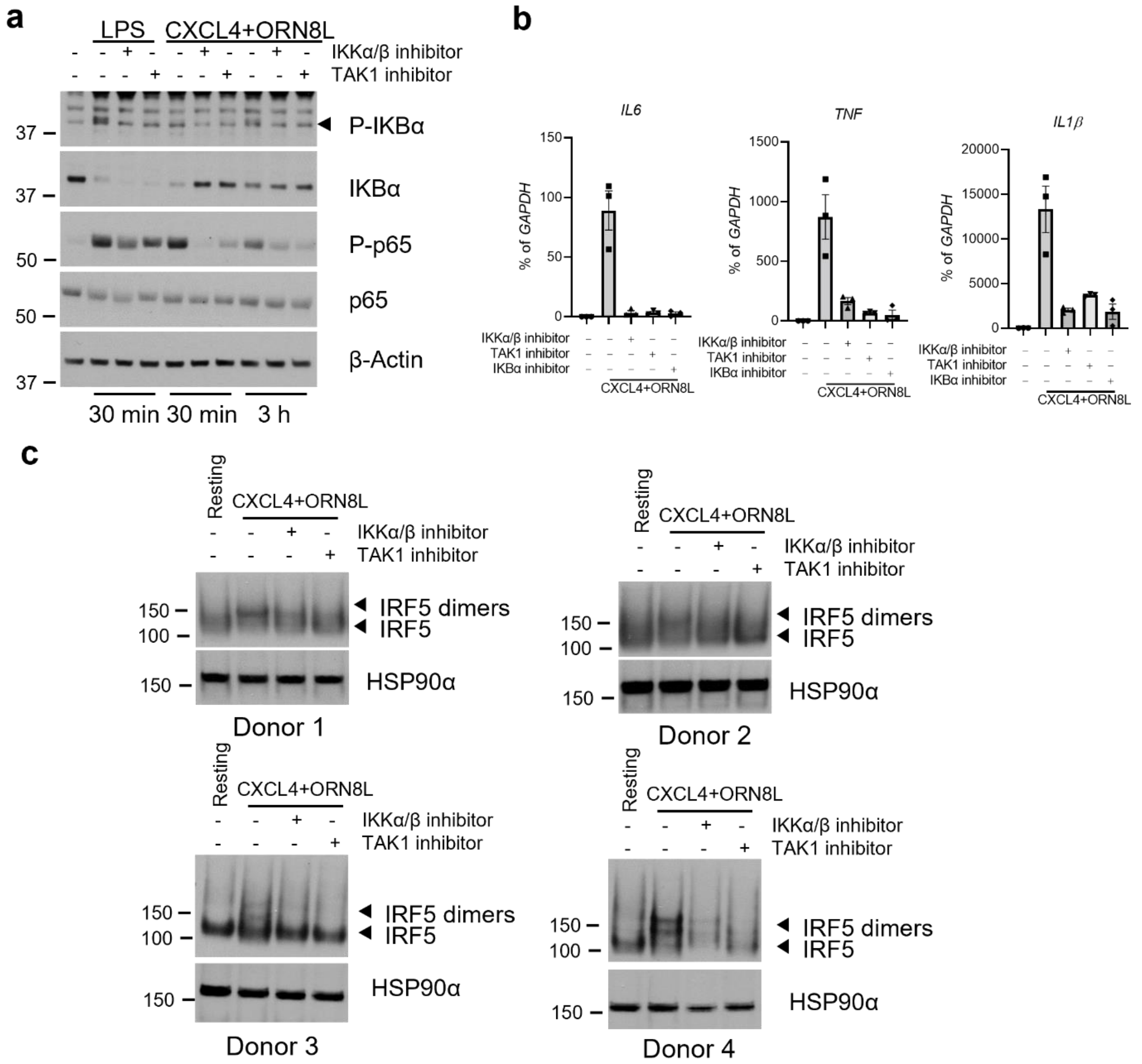

Supplementary Figure 13. (**a**) Immunoblots of phospho-IKBα, total IKBα, and phospho-p65 and total p65 using whole cell lysates from the indicated conditions (data representative of 4 independent experiments). (**b**) mRNA of *IL6*, *TNF* and *IL1β* was measured by qPCR and normalized relative to *GAPDH* mRNA in cells stimulated with CXCL4 and/or ORN8L and IKKα/β inhibitor BMS-345541 (10 μM), TAK1 inhibitor Takinib (10 μM), and IKBα inhibitor Bay 11-7085 (10 μM), respectively (n = 3 donors). (**c**) Immunoblot of IRF5 using whole cell lysates with the indicated conditions run on nondenaturing gels. HSP90α serves as loading control. Four independent experiments are shown. Data are depicted as mean ± SEM for **b**. Source data are provided as a Source Data file.

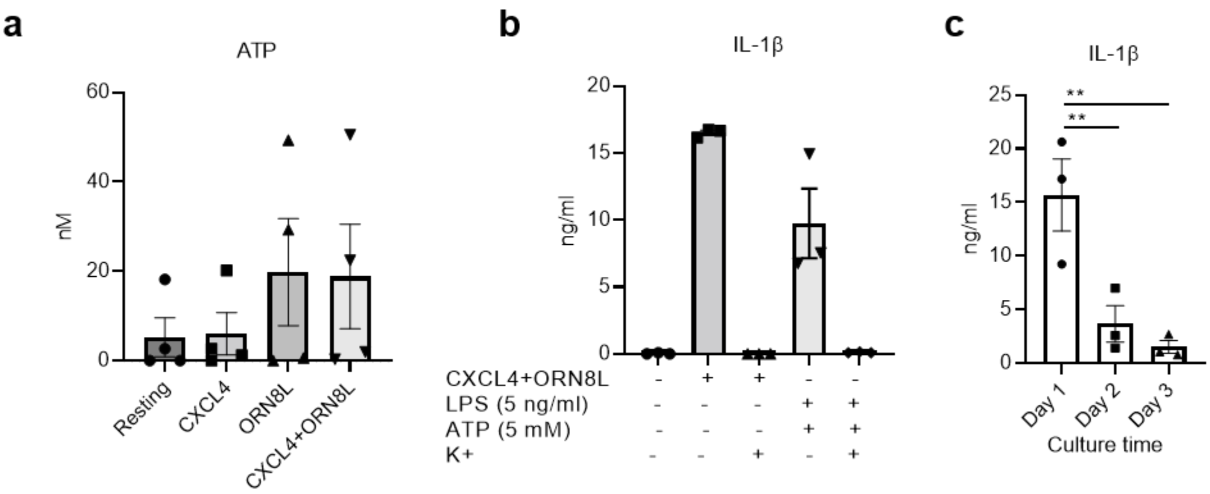

Supplementary Figure 14. (**a**) ATP concentration in indicated cell culture medium detected using ATP Determination Kit (n = 4 donors). (**b**) 130 mM extracellular concentration of K+ suppressed (CXCL4 + ORN8L)-induced IL-1β secretion (n = 3 donors). (**c**) ELISA of IL-1β protein in culture supernatants of monocytes cultured in presence of M-CSF for 1-3 days prior to CXCL4 and TLR8 costimulation for 6h (n = 3 donors). Data is depicted as mean ± SEMdonors; $^{**}p \leq 0.01$ by one-way ANOVA. Source data are provided as a Source Data file.

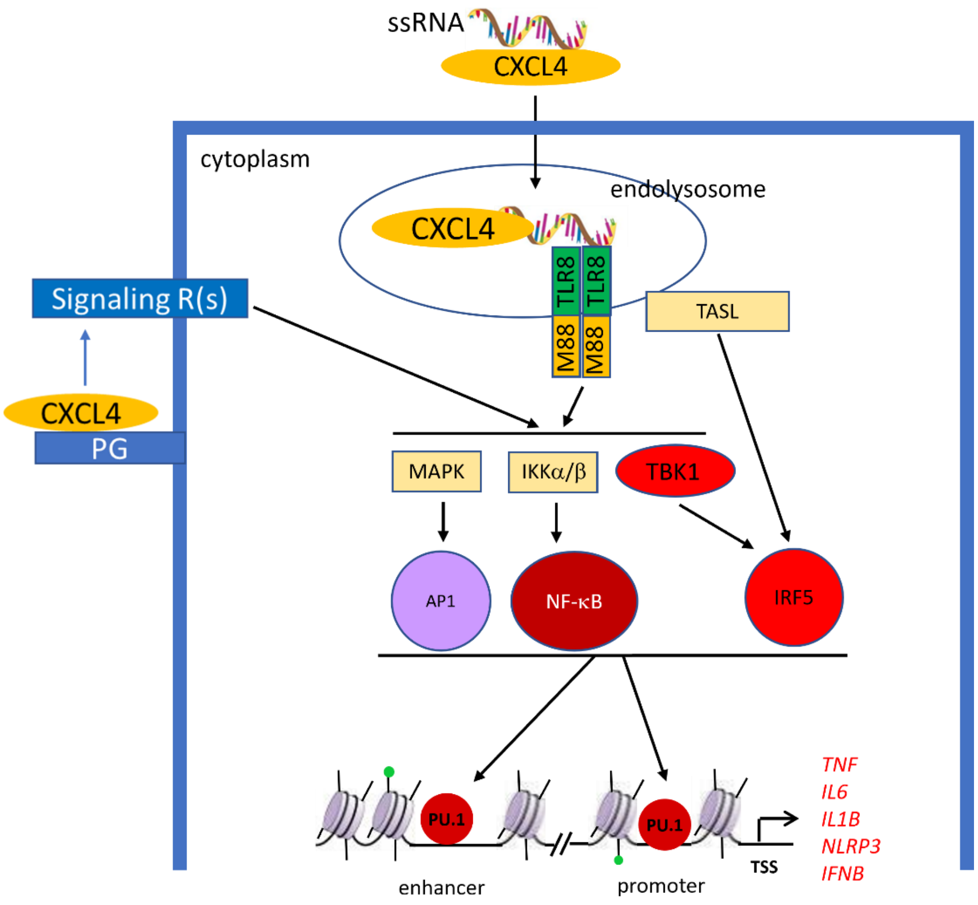

Supplementary Figure 15. A schematic model linking CXCL4 and TLR8 signaling with chromatin remodeling and de novo enhancers associated with inflammatory genes.

Supplementary table 1: Primers and siRNAs.

Primers for qPCR	Source	
Human *GAPDH* F: ATCAAGAAGGTGGTGAAGCA; R: GTCGCTGTTGAAGTCAGAGGA	This paper	
Human *IL6* F: TAATGGGCATTCCTTCTTCT; R: TGTCCTAACGCTCATACTTTT	This paper	
Human *IL12B* F: GGGCACAGATGCCCATTCGCT; R: GGGCACAGATGCCCATTCGCT	This paper	
Human *TNF* F: AATAGGCTGTTCCCATGTAGC; R: AGAGGCTCAGCAATGAGTGA	This paper	
Human *IL6* primary transcript (PT) F: TTGCAAGGAAGGTTTTTGGAG R: CTTGGGTTCAGTTCCAAGCTC	This paper	
Human *IL12B* primary transcript (PT) F: AAGCACTTTGGAGGAAGCATAG; R: TGAACCCAAGTCAATGTGAGTC	This paper	
Human *TNF* primary transcript (PT) F: TAAGGGTGACTCCCTCGATGT; R: CCAAACCCAAACCCAGAATTA	This paper	
Human *IL1β* F: TTCGACACATGGGATAACGAGG; R: TTTTTGCTGTGAGTCCCGGAG	This paper	
Human *CXCL10* F: TTAATCTTGTCTCTGGGCTTGG; R: GTTGGGGAATGAGGTTAGGG	This paper	
Human *IRF1* F: GCACTAAGCGAAAATTGCA; R: GGGAGTTTCCTTCACATTCA	This paper	
Human *NLRP3* F: GATCTTCGCTGCGATCAACAG; R: CGTGCATTATCTGAACCCCAC	This paper	
Mouse *Gapdh* F: ATCAAGAAGGTGGTGAAGCA; R: AGACAACCTGGTCCTCAGTGT	This paper	
Mouse *Il6* F: TGGCTAAGGACCAAGACCATCCAA; R: AACGCACTAGGTTTGCCGAGTAGA	This paper	
Mouse *Tnf* F: CCCTCACACTCAGATCATCTTCT; R: GCTACGACGTGGGCTACAG	This paper	
Mouse *Il1β* F: AGCTTCCTTGTGCAAGTGTCT; R: GACAGCCCAGGTCAAAGGTT	This paper	
**FAIRE Primers for qPCR**		
Human *IL6* (promoter) F: ACCCTCACCCTCCAACAAAG; R: GCAGAATGAGCCTCAGACATC	This paper	
Human *TNF* (promoter) F: GCCCCAGGGACATATAAAGG; R: GCCCCAGGGACATATAAAGG	This paper	
Human *IL12B* (promoter) F: CCCAGAAGGTTTTGAGAGTTGT; R: GATGTTGTTTCTTCTGCTGCTG	This paper	
**Oligonucleotides**	**Source**	**CAS#**
siGENOME Human TBK1 siRNA	Horizon	M-003788-02-0005
siGENOME Human IKBKE siRNA	Horizon	M-003723-02-0005
siGENOME Human IRF5 siRNA	Horizon	M-011706-00-0005
ON-TARGETplus Human MYD88 siRNA	Horizon	L-004769-00-0005

ON-TARGETplus Human TICAM1 siRNA	Horizon	L-012833-00-0005

Supplementary table 2: Antibodies and Others.

Antibodies	Source	CAS#
IkBa (1:1000)	Cell Signaling Technology	9242s
Phospho-p38 MAPK (Thr180/Tyr182) (3D7) (1:1000)	Cell Signaling Technology	9215S
anit-p38 (1:1000)	Cell Signaling Technology	9212S
Phospho-p44/42 MAP Kinase (ERK1/2) (1:1000)	Cell Signaling Technology	9101S
ERK1/2 (1:1000)	Cell Signaling Technology	9102S
TLR8 Polyclonal Antibody (1:500)	Thermofisher Scientific	PA5-80137
TBK1/NAK (E8I3G) (1:1000)	Cell Signaling Technology	38066S
Phospho-TBK1/NAK (Ser172) (D52C2) (1:1000)	Cell Signaling Technology	5483T
Phospho-IKKε (Ser172) (D1B7) (1:500)	Cell Signaling Technology	8766S
IKKε (1:500)	Cell Signaling Technology	2690T
NF-κB p65 (D14E12) (1:1000)	Cell Signaling Technology	8242S
Phospho-NF-κB p65 (Ser536) (93H1) (WB 1:1000; FC 1:1600)	Cell Signaling Technology	3033S
Goat anti-Rabbit IgG (H+L) Cross-Adsorbed Secondary Antibody, Alexa Fluor 594 (1:2000)	Thermofisher Scientific	A-11012
Phospho-IκBα (Ser32/36) (5A5) (1:1000)	Cell Signaling Technology	9246S
IKBα (1:1000)	Cell Signaling Technology	9242S
Phospho-IRF-3 (Ser396) (D6O1M)(1:1000)	Cell Signaling Technology	29047S
IRF-3 (D6I4C) (1:1000)	Cell Signaling Technology	11904T
IRF5 Polyclonal Antibody (1:1000)	Invitrogen	PA5-19504
β-Actin (D6A8) Rabbit mAb (1:5000)	Cell Signaling Technology	8457
NLRP3 (D4D8T) Rabbit mAb (1:250)	Cell Signaling Technology	15101
Human IL-1 beta /IL-1F2 Antibody (2805R) (1 ug/ml)	R&D Systems	MAB601R-100
Caspase-1 (D7F10) Rabbit mAb (1:250)	Cell Signaling Technology	3866
Cleaved Gasdermin D (Asp275) (E7H9G) (1:500)	Cell Signaling Technology	36425
AIM2 (D5X7K) Rabbit mAb (1:500)	Cell Signaling Technology	12948
Human IL-1 beta /IL-1F2 Biotinylated Antibody (0.4 ug/ml)	R&D Systems	BAF201
PE Streptavidin (1:400)	Biolegend	405203
Phospho-IRF-3 (Ser386) (E7J8G) XP® Rabbit mAb (Alexa Fluor® 488 Conjugate) (1:50)	Cell Signaling Technology	73981
CD11c Hamster anti-Mouse, BUV737, Clone: N418 (1:200)	BD Biosciences	BDB749039
Pacific Blue™ anti-mouse Ly-6C Antibody (HK1.4) (1:200)	Biolegend	128014
PerCP/Cyanine5.5 anti-mouse CD206 (MMR) Antibody (C068C2) (1:100)	Biolegend	141716
Human/Primate IL-6 Antibody (6708) (4 ug/ml)	R&D Systems	MAB206-SP
Human/Primate IL-6 Biotinylated Antibody (0.4 ug/ml)	R&D Systems	BAF206
Human TNF-alpha Antibody (28401) (4 ug/ml)	R&D Systems	MAB610-SP
Human TNF-alpha Biotinylated Antibody (0.4 ug/ml)	R&D Systems	BAF210
Human IL-10 R alpha Antibody (37607) (10 ug/ml)	R&D Systems	MAB274-100

Human IL-10 Antibody (23738) (10 ug/ml)	R&D Systems	MAB217-100
**TLR ligands, inhibitors and recombinant proteins**		
Recombinant Human IL-1 beta/IL-1F2 Protein	R&D Systems	201-LB-005
LPS	Invivogen	TLRL-3pelps
PAM3CYS	Invivogen	TLRL-PMS
Poly I:C	Invivogen	TLRL-PIC
ORN8L	Chemgenes Corporation	(Lan et al., 2007)
ORN8L-AF488	Chemgenes Corporation	
Recombination Human CXCL4	PEPROTECH	300-16
PF-4 (CXCL4) human	Sigma-Aldrich	SRP3142
Recombinant Human IL-6	Peprotech	200-06
Recombinant Human TNF-alpha	Peprotech	300-01A
Pertussis Toxin	R&D Systems	3097/50U
SCH 202676 hydrobromide	R&D Systems	1400/10
BMS-345541-IKKα/β inhibitor	Selleckchem	S8044
Bay 11-7085-IKBα inhibitor	Selleckchem	S7352
Takinib	Selleckchem	S8663-5mg
NLRP3 Inhibitor, MCC950	Sigma Aldrich	5381200001
Ac-YVAD-cmk, CASP1 inhibitor	Sigma Aldrich	SML0429
SB202190, Hydrochloride, p38 inhibitor	Calbiochem	559393
Bafilomycin A1	MCE	HY-100558
MRT67307 HCl (dual IKKϵ and TBK1 inhibitor)	Selleckchem	S7948
TBK1/IKKε-IN-2	MCE	HY-12453
GSK8612-TBK1 inhibitor	Selleckchem	S8872
AMG 487 CXCR3 antagonist	TOCRIS	4487
JNK Inhibitor II	Sigma Aldrich	420119
U0126 MEK1/2 inhibitor	Sigma Aldrich	662005
