# Supplementary material for: CXCL4 synergizes with TLR8 for TBK1-IRF5 activation, epigenomic remodeling and inflammatory response in human monocytes
Source: Nat Commun. 2022 Jun 14;13:3426. doi: 10.1038/s41467-022-31132-7 (PMC9195402; doi:10.1038/s41467-022-31132-7)
Supplement: Supplementary file 3 — Description of Additional Supplementary Files [file 41467_2022_31132_MOESM3_ESM.pdf]

Description of Additional Supplementary Files

Supplementary Data 1. Transcription factor binding identified by differential binding analysis of n = 1011 JASPAR motifs by TOBIAS using BINDetect algorithm in the conditions of CXCL4, ORN8L and (CXCL4 + ORN8L) stimulation.

Supplementary Data 2. Transcription factor motifs identified by HOMER de novo motif analysis of C1-C7 peaks from **Figure 5b**.
