# Supplementary material for: CXCL4 synergizes with TLR8 for TBK1-IRF5 activation, epigenomic remodeling and inflammatory response in human monocytes
Source: Nat Commun. 2022 Jun 14;13:3426. doi: 10.1038/s41467-022-31132-7 (PMC9195402; doi:10.1038/s41467-022-31132-7)
Supplement: Supplementary file 6 — Reporting Summary [file 41467_2022_31132_MOESM6_ESM.pdf]

nature portfolio

Corresponding author(s): Lionel B. Ivashkiv

Last updated by author(s): May 23, 2022

Reporting Summary

Nature Portfolio wishes to improve the reproducibility of the work that we publish. This form provides structure for consistency and transparency in reporting. For further information on Nature Portfolio policies, see our Editorial Policies and the Editorial Policy Checklist.

Statistics

For all statistical analyses, confirm that the following items are present in the figure legend, table legend, main text, or Methods section.

n/a	Confirmed	
☐	☒	The exact sample size (*n*) for each experimental group/condition, given as a discrete number and unit of measurement
☐	☒	A statement on whether measurements were taken from distinct samples or whether the same sample was measured repeatedly
☐	☒	The statistical test(s) used AND whether they are one- or two-sided<br>*Only common tests should be described solely by name; describe more complex techniques in the Methods section.*
☒	☐	A description of all covariates tested
☐	☒	A description of any assumptions or corrections, such as tests of normality and adjustment for multiple comparisons
☐	☒	A full description of the statistical parameters including central tendency (e.g. means) or other basic estimates (e.g. regression coefficient) AND variation (e.g. standard deviation) or associated estimates of uncertainty (e.g. confidence intervals)
☐	☒	For null hypothesis testing, the test statistic (e.g. *F*, *t*, *r*) with confidence intervals, effect sizes, degrees of freedom and *P* value noted<br>*Give P values as exact values whenever suitable.*
☒	☐	For Bayesian analysis, information on the choice of priors and Markov chain Monte Carlo settings
☒	☐	For hierarchical and complex designs, identification of the appropriate level for tests and full reporting of outcomes
☒	☐	Estimates of effect sizes (e.g. Cohen's *d*, Pearson's *r*), indicating how they were calculated

Our web collection on statistics for biologists contains articles on many of the points above.

Software and code

Policy information about availability of computer code

Data collection	Flow cytometry data was collected using BD FACSymphony™ A3 Cell Analyzer and Flowjo software. RNAseq data was processed using FastQC, cutadapt, STAR aligner and edgeR. These are open source.
Data analysis	Flowjo V10 was used for FACS data analysis.<br>Pathway analysis for RNA seq data was conducted using IPA<br>ATAC seq for motif analysis was conducted using either HOMER or TOBIAS.<br>Graphpad Prism was used for data analysis.

For manuscripts utilizing custom algorithms or software that are central to the research but not yet described in published literature, software must be made available to editors and reviewers. We strongly encourage code deposition in a community repository (e.g. GitHub). See the Nature Portfolio guidelines for submitting code & software for further information.

Data

Policy information about availability of data

All manuscripts must include a data availability statement. This statement should provide the following information, where applicable:

- Accession codes, unique identifiers, or web links for publicly available datasets
- A description of any restrictions on data availability
- For clinical datasets or third party data, please ensure that the statement adheres to our policy

The RNAseq and ATACseq data sets that support the findings of this study and were generated by the authors as part of this study have been deposited in the Gene

Expression Omnibus database with the accession code: GSE181891. The code GSE181891 is composed of the following subseries: GSE181886 for ATAC-seq data of distinct regulation of gene promoters and enhancers by CXCL4 and TLR8 in human CD14+ monocytes; GSE181889 for quantitative analysis of CXCL4 and TLR8 signaling crosstalk in human primary monocytes by RNA-Seq. For the other data, a Source Data File is provided with this paper.

Field-specific reporting

Please select the one below that is the best fit for your research. If you are not sure, read the appropriate sections before making your selection.

☒ Life sciences ☐ Behavioural & social sciences ☐ Ecological, evolutionary & environmental sciences

For a reference copy of the document with all sections, see nature.com/documents/nr-reporting-summary-flat.pdf

Life sciences study design

All studies must disclose on these points even when the disclosure is negative.

Sample size	No statistical methods were used to predetermine sample size. Sample size was based on our previous publications and experience.
Data exclusions	No data were excluded from analysis
Replication	The experiments were repeated at least 3 times except for Fig 3c which has n = 2 at multiple time points and a large effect size, and Fig S6b which is confirmatory of previous reports that CXCL4 does not work via G protein coupled receptors in myeloid cells. Figs. 2a (n = 4) and 2b (n = 2) cumulatively represent 6 independent experiments testing the same experimental conditions. The exact number of repeats is noted in the figure legends. Replication was successful for all of the reported experiments.
Randomization	Blood products were from random anonymous blood donors and were purchased from the New York Blood Center. Cells from each donor were randomly allocated into experimental groups.
Blinding	The investigators were not blinded to the treatment conditions which was not required given the in vitro experimental design and quantitative impartial outcomes that did not require subjective decision making.

Reporting for specific materials, systems and methods

We require information from authors about some types of materials, experimental systems and methods used in many studies. Here, indicate whether each material, system or method listed is relevant to your study. If you are not sure if a list item applies to your research, read the appropriate section before selecting a response.

Materials & experimental systems

n/a	Involved in the study	
☐	☒	Antibodies
☒	☐	Eukaryotic cell lines
☒	☐	Palaeontology and archaeology
☐	☒	Animals and other organisms
☐	☒	Human research participants
☒	☐	Clinical data
☒	☐	Dual use research of concern

Methods

n/a	Involved in the study	
☒	☐	ChIP-seq
☐	☒	Flow cytometry
☒	☐	MRI-based neuroimaging

Antibodies

Antibodies used

Antibodies Source CAS#
IkBa (1:1000) Cell Signaling Technology 9242s
Phospho-p38 MAPK (Thr180/Tyr182) (3D7) (1:1000) Cell Signaling Technology 9215S
anit-p38 (1:1000) Cell Signaling Technology 9212S
Phospho-p44/42 MAP Kinase (ERK1/2) (1:1000) Cell Signaling Technology 9101S
ERK1/2 (1:1000) Cell Signaling Technology 9102S
TLR8 Polyclonal Antibody (1:500) Thermofisher Scientific PA5-80137
TBK1/NAK (E8I3G) (1:1000) Cell Signaling Technology 38066S
Phospho-TBK1/NAK (Ser172) (D52C2) (1:1000) Cell Signaling Technology 5483T
Phospho-IKKε (Ser172) (D1B7) (1:500) Cell Signaling Technology 8766S
IKKε (1:500) Cell Signaling Technology 2690T
NF-κB p65 (D14E12) (1:1000) Cell Signaling Technology 8242S
Phospho-NF-κB p65 (Ser536) (93H1) (1:1000) Cell Signaling Technology 3033S
Goat anti-Rabbit IgG (H+L) Cross-Adsorbed Secondary Antibody, Alexa Fluor 594 (1:2000) Thermofisher Scientific A-11012
Phospho-IκBα (Ser32/36) (5A5) (1:1000) Cell Signaling Technology 9246S

IKBα (1:1000) Cell Signaling Technology 9242S
Phospho-IRF-3 (Ser396) (D6O1M) (1:500) Cell Signaling Technology 29047S
IRF-3 (D6I4C) (1:1000) Cell Signaling Technology 11904T
IRF5 Polyclonal Antibody (1:1000) Invitrogen PA5-19504
β-Actin (D6A8) Rabbit mAb (1:5000) Cell Signaling Technology 8457
NLRP3 (D4D8T) (1:250) Rabbit mAb Cell Signaling Technology 15101
Human IL-1 beta /IL-1F2 Antibody (2805R) (1 ug/ml)R&D Systems MAB601R-100
Caspase-1 (D7F10) (1:250)Rabbit mAb Cell Signaling Technology 3866
Cleaved Gasdermin D (Asp275) (E7H9G) (1:500)Cell Signaling Technology 36425
AIM2 (D5X7K) Rabbit mAb (1:500) Cell Signaling Technology 12948
Human IL-1 beta /IL-1F2 Biotinylated Antibody (0.4 ug/ml) &D Systems BAF201
PE Streptavidin (1:400) Biolegend 405203
Phospho-IRF-3 (Ser386) (E7J8G) XP® Rabbit mAb (Alexa Fluor® 488 Conjugate) (1:50) Cell Signaling Technology 73981
CD11c Hamster anti-Mouse, BUV737, (1:200) Clone: N418 BD Biosciences BDB749039
Pacific Blue™ anti-mouse Ly-6C Antibody (HK1.4) (1:200)Biolegend 128014
PerCP/Cyanine5.5 anti-mouse CD206 (MMR) Antibody (C068C2) (1:100) Biolegend 141716
Human/Primate IL-6 Antibody (6708) (4 ug/ml) R&D Systems MAB206-SP
Human/Primate IL-6 Biotinylated Antibody (0.4 ug/ml) R&D Systems BAF206
Human TNF-alpha Antibody (28401) (4 ug/ml) R&D Systems MAB610-SP
Human TNF-alpha Biotinylated Antibody (0.4 ug/ml) R&D Systems BAF210
Human IL-10 R alpha Antibody (37607) (10 ug/ml) R&D Systems MAB274-100
Human IL-10 Antibody (23738) (10 ug/ml) R&D Systems MAB217-100

Validation

Validation was performed by the source company. Additional validation of IRF5 antibodies was performed by us using siRNA.

Animals and other organisms

Policy information about studies involving animals; ARRIVE guidelines recommended for reporting animal research

Laboratory animals

Male C57BL/6J mice (Strain #:000664) at 6 to 8 weeks old were purchased from the Jackson Laboratories and housed under specific pathogen-free conditions.

Wild animals

Study did not involve wild animals

Field-collected samples

Study did not involve animals collected from the field.

Ethics oversight

Animal experiments were approved by the Weill Cornell Medicine IACUC Committee.

Note that full information on the approval of the study protocol must also be provided in the manuscript.

Human research participants

Policy information about studies involving human research participants

Population characteristics

Anonymous blood products were purchased from the New York Blood Center and used for in vitro experiments. Although this does not constitute human subjects research as per PHS SF424 we are disclosing this for full transparency.

Recruitment

N/A

Ethics oversight

The study was approved by the Hospital for Special Surgery IRB.

Note that full information on the approval of the study protocol must also be provided in the manuscript.

Flow Cytometry

Plots

Confirm that:

- [x] The axis labels state the marker and fluorochrome used (e.g. CD4-FITC).
- [x] The axis scales are clearly visible. Include numbers along axes only for bottom left plot of group (a 'group' is an analysis of identical markers).
- [x] All plots are contour plots with outliers or pseudocolor plots.
- [x] A numerical value for number of cells or percentage (with statistics) is provided.

Methodology

Sample preparation

1. p65 phosphorylation. Cells were harvested from cultured dishes and fixed with 4% PFA for 15 min at RT, then

permeabilized with 90% cold methanol for 10 min on ice. After washing, cells were stained with phospho-p65 antibody for 1 h at RT and then secondary AF594-conjugated antibody for 30 min at RT.
2. ORN8L uptake in human monocytes. 5 x 105 human monocytes were plated in 48 well plates and incubated with fluorescently labeled ORN8L (ORN8L-AF488; obtained from Chemgenes Corporation) with/without human CXCL4 for the times indicated in the figure legend. Cells then were harvested, washed with FACS buffer and analyzed by flow cytometry and the MFI of ORN8L-AF488 was analyzed using flowjo software.
3.Flow cytometry (FACS) analysis. Immature floating BMDC were harvested, stained with Fixable Viability Dye eFluor™ 780 (Thermo Fisher Scientific, 65-0865-18), and then anti-CD11C (BD Biosciences, 749039), anti-Ly6C (Biolegend, 128014) and anti-CD206 (Biolegend, 141716) antibodies for 30 min at 4 degree. After washing, the cells were analyzed using BD FACSymphony™ A3 Cell Analyzer and Flowjo software.

Instrument

BD Symphony a3

Software

Flowjo

Cell population abundance

There was no sorting performed. 100,000-200,000 cells were used for staining.

Gating strategy

The experiments analyzed a single cell population using one primary antibody. Gating strategy is provided in Supplementary Fig 7a.

☒ Tick this box to confirm that a figure exemplifying the gating strategy is provided in the Supplementary Information.
